# Superior Labial Artery Mucosal Flap (SLAM) for Reconstruction of Major Lower Lip Mucosal Defects

**DOI:** 10.1002/hed.70373

**Published:** 2026-06-22

**Authors:** Nicholas Shannon, Takako Eva Yabe, Bruce Ashford

**Affiliations:** ^1^ Department of Head and Neck Surgery Illawarra Shoalhaven Local Health District, Wollongong Hospital Wollongong New South Wales Australia; ^2^ Graduate School of Medicine University of Wollongong Wollongong New South Wales Australia

**Keywords:** head and neck cancer, lower lip, reconstruction, squamous cell carcinoma, surgical technique

## Abstract

**Background:**

The superior labial artery mucosal (SLAM) flap is a versatile, pedicled axial flap ideally suited for mucosal lining in lower lip reconstruction.

**Methods:**

This article details the surgical anatomy and technique of the SLAM flap, highlighting its role as a standalone liner or mucosal adjunct to microvascular free tissue transfer in major composite reconstructions.

**Results:**

Clinical success relies on preserving the submucosal venous plexus, with the axial blood supply from the superior labial artery providing resilience against prior radiation therapy. By providing pliable, homologous mucosa, the SLAM flap effectively restores oral competence and defines the vermilion border.

**Conclusions:**

The SLAM flap is a highly reliable option for internal lining in complex lower lip defects.

## 1 Introduction

The lower lip is a dynamic, trilaminar structure of sensate skin, sphincteric orbicularis oris muscle (OOM), and mucosal lining that is essential for oral competence, speech articulation, and facial expression. Ablative oncologic surgery disrupts this trilaminar complex; reconstruction must therefore prioritize functional restoration over simple volume replacement. Reconstructive efforts must secure an adequate watertight seal to prevent functional sequelae such as drooling and microstomia.

Functional goals include lip competence; achieving a dynamic sphincter capable of sealing itself effectively to prevent drooling and nutritional impairment, and recreating the labial mucogingival gutter: allowing for natural movement of the cheek and tongue and proper seating of dentures. Aesthetic success hinges on minimizing distortion of the natural lip volume and contour, avoiding microstomia (overly narrow oral stoma), and ensuring the vermilion border is precisely reconstructed. Although external skin and muscular bulk are often the focus, the restoration of a thin, pliable, and vascularized mucosal lining is crucial for creating a watertight seal, maintaining internal moisture, and defining the vermilion.

### 1.1 Major Lower Lip Defects

Major lip defects (50%–75% involvement) require local tissue transfer techniques such as local advancement flaps (Webster‐Modified Bernard or Karapandzic flap) or lip switch techniques (Abbe or Estlander flaps) often suffice to restore full‐thickness structure [1, 2]. However, near‐total or total lip loss often necessitates microvascular free tissue transfer (e.g., Radial Forearm Free Flap [RFFF] or Anterolateral Thigh [ALT] flap) to supply adequate external bulk and static support [3]. Relying solely on these flaps frequently results in suboptimal internal lining. The challenge in total lip reconstruction is finding a pliable, resilient mucosal match for the internal surface, a goal difficult to achieve with folded skin paddles, non‐epithelialized muscle flaps, or non‐vascularized grafts.

To address this critical lining deficit, local mucosal flaps based on the highly reliable facial artery (FA) have emerged. The superior labial artery mucosal (SLAM) flap, a specific variant of the FA musculomucosal (FAMM) family, delivers the mucosal lining required for the posterior surface of a composite reconstruction, addressing the complexity of internal mucosal defects directly.

### 1.2 The FAMM Flap Family

Pribaz et al. first described the FAMM flap for intraoral reconstruction [4]. The FAMM flap is a versatile, pedicled composite flap derived from the buccal mucosa and underlying buccinator/OOM complex. It is suitable for small‐to‐medium‐sized defects throughout the oral cavity [5].

The SLAM flap is a specific variant within this family, defined by its blood supply derived from its namesake artery. The SLAM flap is harvested from the upper lip mucosa. While previous descriptions focus on the nose [6], floor of mouth [7], and buccal defects [8], this mucosa is equally suited and ideal for vermilion and lip lining. Its consistent vascularity and tissue characteristics distinguish it as a preferred local option for internal lip coverage.

#### 1.2.1 Surgical Anatomy and Physiological Basis of the SLAM Flap

##### 1.2.1.1 Arterial Supply

The FA originates from the external carotid artery and follows a tortuous course in the face. Proximal to the oral commissure, the FA typically resides in the dense fibrous tissue near the corner of the mouth, continuing along the superior border of the upper lip in the buccal region. The vessel generally lies within 20 mm (with an average of 16 mm) from the labial commissure [9].

The FA, after following its tortuous course, gives rise to the superior labial artery (SLA) and the Inferior Labial Artery (ILA) just proximal to the oral commissure. These vessels course in the submucosa, in a plane situated between the mucosa and the OOM [10]. This relationship enables the reliable harvest of a vascularized mucosal or musculomucosal paddle.

With an average length 45.4 mm [7], the SLA provides sufficient pedicle length for the flap to reach the contralateral commissure or the floor of the mouth. This reach is augmented by the robust anastomosis of the SLA with its contralateral counterpart, typically near the philtrum, which allows for retrograde transposition (superiorly based) of the flap [7]. For total lower lip reconstruction, a bilateral SLAM flap harvest is preferred, effectively doubling the mucosal surface area.

##### 1.2.1.2 Venous Drainage

Venous outflow remains the primary technical challenge and determines the complication profile of the SLAM flap. The flap relies not on a single vena comitans, but on a rich submucosal venous plexus for drainage [9]. Although the facial vein may be sacrificed without immediate flap failure [11]; careful surgical execution is required to safeguard the integrity of this crucial venous plexus. Plexus outflow obstruction, caused by pedicle kinking, excessive tension, or a narrow base, compromises flap viability.

Venous stasis, rather than arterial occlusion, remains the chief circulatory risk, with clinical monitoring focused on rapid darkening or swelling of the mucosal paddle. Initial management involves the immediate release of any constricting tacking sutures and assessment of kinking or tension at the tunnel site. If congestion persists despite mechanical relief, medicinal leech therapy ( *Hirudo medicinalis* ), pharmacological equivalents (heparinized saline soaks or systemic anticoagulants), or chemical decompression (e.g., topical nitroglycerin) can be employed as a bridge to allow for the development of neoangiogenesis and secondary venous channels, typically over 3–5 days.

### 1.3 Flap Design and Harvest

#### 1.3.1 Preoperative Planning and Assessment

Preoperative planning begins with a clinical assessment of the donor site, focusing on mucosal laxity and the presence of any scarring from prior interventions. Manual palpation of the FA as it traverses the mandibular notch provides an initial landmark and a handheld 8–10 MHz doppler probe should be used to definitively map out the artery's course towards the oral commissure and its continuation as the SLA, as well as confirming SLA patency.

Pre‐operative color duplex ultrasound can be considered in patients with complex histories involving previous radiation or extensive surgery. Surgeons must exclude patients with a compromised or absent FA, as this remains the primary contraindication. Patients should receive appropriate antimicrobial prophylaxis targeting the flora of the oral cavity.

Local infiltration of vasoconstrictive agents, such as adrenaline, around the planned flap outline should be strictly avoided. Vasoconstrictive agents are avoided to prevent the risk of inducing vasospasm in the delicate FA, which could lead to complications in identification and dissection, compounding the vulnerability inherent in the flap's venous drainage system.

#### 1.3.2 Flap Design and Marking

Flap design must satisfy three primary constraints to ensure donor site function and flap viability (Table 1). First, the anterior incision must be positioned a minimum of 1.0 cm posterior to the labial commissure [9]. Preserving this margin prevents donor site contraction, minimizing the risk of oral stoma distortion and the aesthetic and functional complications of microstomia. Second, the maximum flap width is limited to 2.0 cm if the surgeon wishes to complete a primary, tension‐free, multilayered donor site closure [7]. The harvest site does not mandate primary closure and mucosalization of the defect can occur by secondary intention; if a bilateral design is used, a mucosal bridge should be left at the center of the lip to reduce the risk of stenosis and microstomia. Finally, the pedicle base requires a minimum width of 1.5 cm to safeguard the submucosal venous plexus and ensure adequate outflow [9].

**TABLE 1:** Anatomic constraints and technical specifications for the SLAM flap.

Anatomic constraint	Technical parameters	Surgical rationale
Pedicle artery	Superior labial artery (SLA)	Axial blood flow for high reliability and radiation resistance
Flap composition	Mucosa, submucosa, ± partial thickness orbicularis oris fibers	Provides pliability, bulk, and essential *wet* mucosal surface
Maximum flap width	Max 2.0 cm	Ensure tension‐free donor site closure
Anterior incision limit	1.0 cm posterior to commissure	Avoid distortion, preserve modiolus, and prevent microstomia
Pedicle base width	At least 1.5 cm	Crucial for ensuring adequate venous drainage via the submucosal plexus
Maximum flap length	Max 4.5 cm	Preserve adequate inflow and prevent distal flap ischemia

#### 1.3.3 Flap Harvest and Pedicle Dissection

The mucosal incisions are carefully deepened through the submucosa and may include part of the OOM. Harvesting the flap in this musculomucosal fashion provides additional bulk compared to a simple mucosal graft.

The pedicle dissection requires isolation of the SLA with preservation of the venous plexus. Although the artery may need to be skeletonized to achieve maximum reach, trauma to the vessel walls or undue manipulation of the venous plexus must be avoided to prevent immediate or delayed congestion. Following harvest, the donor site is closed in multiple layers by re‐approximating the muscle and mucosal layers to minimize tension and prevent the inward rolling of the lip vermilion.

In cases where primary closure of the donor site would lead to significant vertical shortening or distortion of the upper lip aesthetics, the donor defect may be left to heal by secondary intention. Initial re‐epithelialization of the donor site typically occurs within 14 days, with full mucosal maturation achieved by 4 weeks. Post‐operative management for the open donor site involves the application of a thin layer of topical antibiotic ointment or petroleum jelly to maintain a moist environment and prevent crusting. Patients are advised to follow a soft diet and perform gentle saline oral rinses after meals. Healing by secondary intention avoids the “purse‐string” deformity associated with primary closure, thereby preserving the native craniocaudal height and natural contour of the upper lip.

#### 1.3.4 Flap Transposition and Inset

The harvested SLAM flap is transposed to the lower lip defect, either directly as a staged flap or via a subcutaneous or submucosal tunnel as an islanded flap. For a staged flap, the flap is transposed over the oral commissure, leaving the vascular pedicle exposed and requiring a second stage, typically 2–3 weeks later, to divide the pedicle and complete the inset. To avoid a second stage, the pedicle can be islanded by skeletonizing the SLA and its associated venous plexus, though preserving a cuff of perivascular tissue to ensure viability of the plexus. This allows the tissue to pass through a submucosal tunnel directly into the defect. While more efficient, this approach carries an increased risk of vessel kinking or mechanical trauma during tunnel passage which can lead to flap compromise.

The inset is orientated with the mucosal surface inwards to create the new internal lining and, if designed appropriately, defining the new vermilion border. When managing major defects with external free flap coverage (e.g., RFFF), the SLAM flap functions as the recipient mucosal interface for the deep margin of the external skin paddle. If a folded free flap design is used, a strip of skin can be de‐epithelialized to onlay the SLAM flap.

#### 1.3.5 Post‐Operative Management

Post‐operative management focuses primarily on preventing mechanical stress and early detection of vascular compromise. Patients should be maintained on a strict full liquid or pureed diet for at least 2 weeks to minimize the activity of the orbicularis muscle and reduce tension across the newly reconstructed mucosal interface. Oral hygiene is required with regular dilute chlorhexidine or saline rinses to manage bacterial load, especially in cases where the upper lip donor site is left to heal by secondary intention. Clinical monitoring must be vigilant for signs of venous congestion, such as rapid swelling or a cyanotic hue, which may indicate compression at the pedicle base. In the subacute phase, once the primary suture lines are stable, patients can begin gentle range‐of‐motion exercise to prevent microstoma and optimize the functional pucker of the lip. If the reconstruction is part of a staged approach, the pedicle is typically divided and contoured at approximately 3 weeks, provided the flap has successfully integrated with the recipient bed's peripheral blood supply. Long‐term follow‐up should prioritize the assessment of vermilion symmetry and the monitoring of any contracture that might necessitate secondary debulking or minor commissuroplasty.

## 2 Clinical Application in Major Lower Lip Mucosal Reconstruction

### 2.1 Indications for the SLAM Flap in the Lower Face

The SLAM flap is a highly versatile option for reconstructing small‐to‐medium intraoral defects, including localized vermilion defects (Figure 1), soft palate fistulas, and defects in the floor of the mouth or tongue. Its capacity to provide thin, pliable, vascularized tissue makes it highly successful in these primary indications.

**FIGURE 1:**
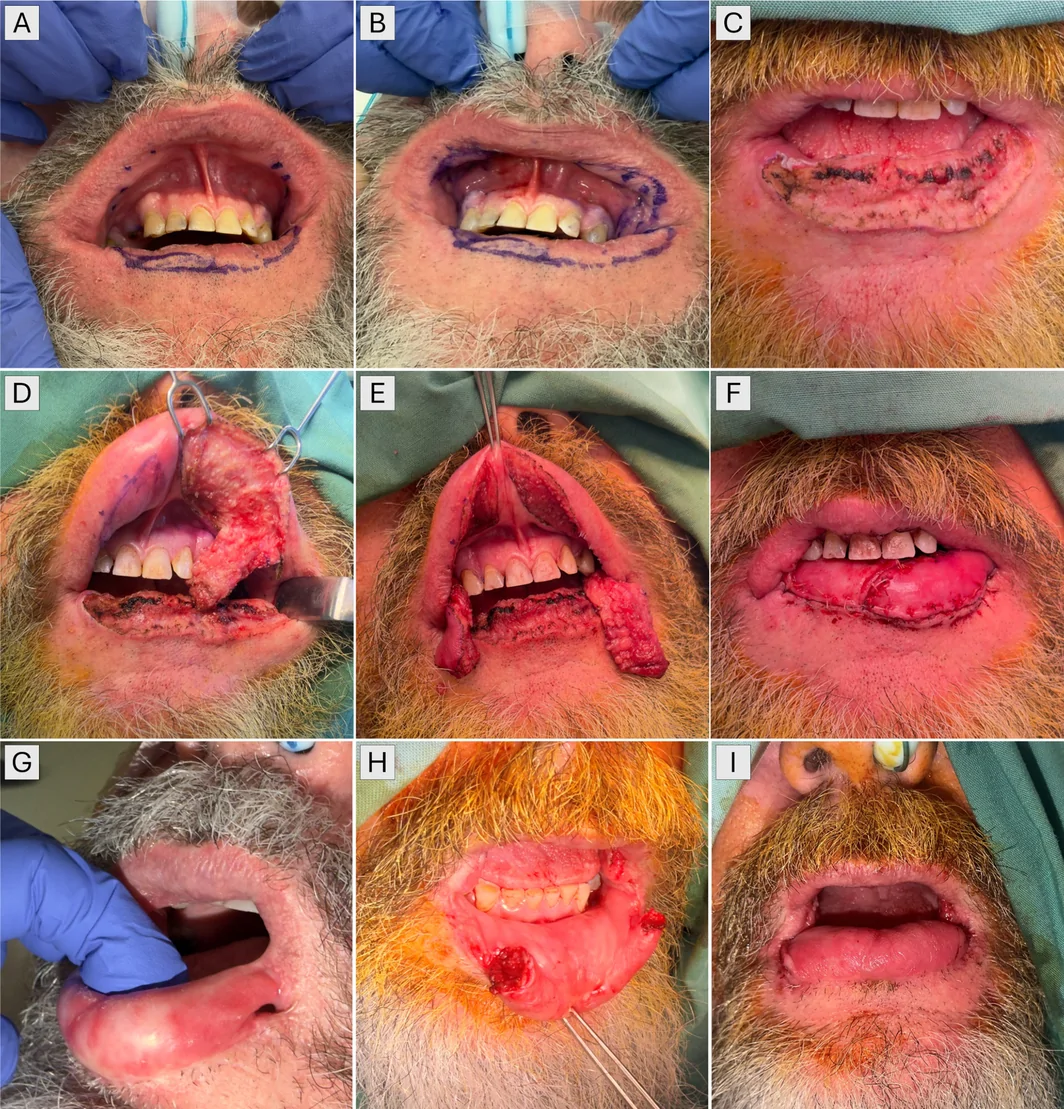
Bilateral pedicled SLAM flap for lower lip vermilion reconstruction in a patient with lower lip SCC. (A) Preoperative markings outlining the course of the SLA and the lower lip lesion. (B) Detailed marking of the bilateral SLAM flap design. (C) Lower lip defect following wide local excision. (D) Elevation of the left SLAM flap. (E) Bilateral flaps raised, showing the preserved mucosal bridge between the donor sites. (F) Immediate postoperative result demonstrating offset medial suture line to mitigate scar contracture. (G) Post‐operative appearance at 15 weeks; the delay in second‐stage division was due to institutional scheduling constraints. (H) Division of vascular pedicles. (I) Final appearance following second‐stage pedicle division and mucosal inset, noting preserved height of the lip vermilion in comparison to pre‐harvest in (C). Abbreviations: SLA, superior labial artery; SLAM, superior labial artery mucosal; SCC, squamous cell carcinoma. [Color figure can be viewed at wileyonlinelibrary.com]

The SLAM flap is particularly useful in composite near total lower lip reconstructions (Figure 2), providing the mucosal lining that guarantees oral competence and appropriate vermilion definition.

**FIGURE 2:**
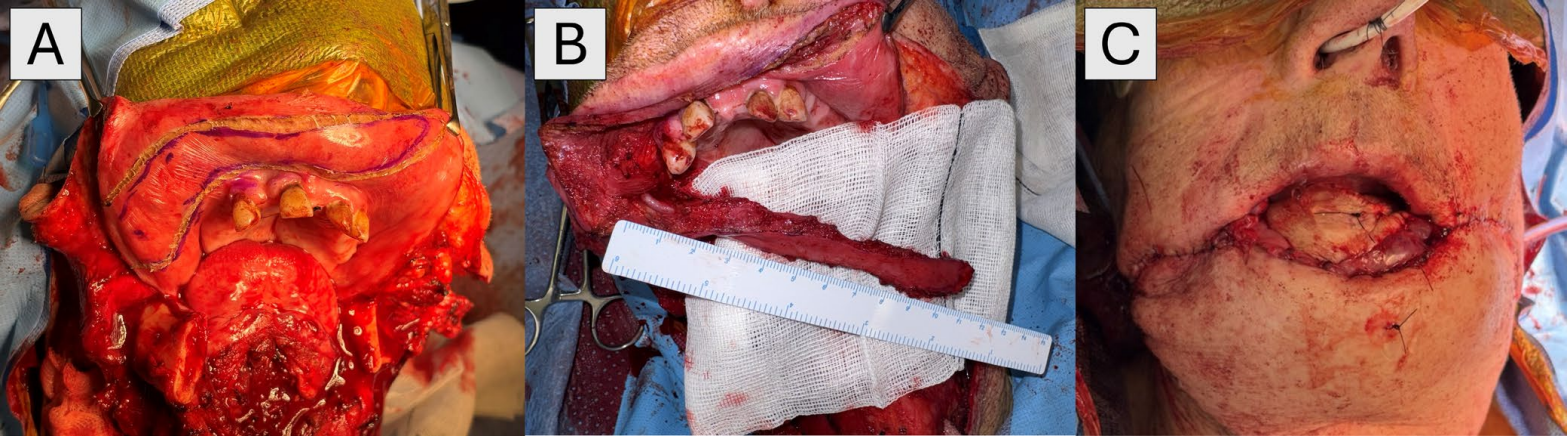
Unilateral SLAM flap utilized in a composite total lower lip reconstruction. The patient presented with recurrent, locally advanced lower lip SCC involving the mandible following radiation therapy and immunotherapy. A unilateral flap design was chosen because the left facial artery was sacrificed during the resection. (A) View of the composite defect and markings for the right‐sided SLAM flap. (B) The mobilized SLAM flap prior to inset. (C) Completed reconstruction integrating a free fibula flap (providing mandible reconstruction and intra‐oral lining), an ALT free flap (providing external skin coverage), and the SLAM flap (reconstructing the lower lip vermilion). Abbreviations: ALT, anterolateral thigh; SCC, squamous cell carcinoma; SLAM, superior labial artery mucosal. [Color figure can be viewed at wileyonlinelibrary.com]

For extensive ablative defects of the lower lip (e.g., 75% loss), a composite approach combining local lining with distant bulk may be required. Free tissue transfer, such as the RFFF, is frequently used to provide the external skin, bulk, and static support (via the Palmaris Longus tendon sling). Although an RFFF provides necessary bulk, its skin paddle offers a suboptimal mucosal interface [12]. By combining the RFFF with a SLAM flap, the surgeon replaces skin‐on‐mucosa closure with a mucosa‐on‐mucosa seal, significantly improving oral competence.

### 2.2 Utility in Previously Radiated Defects

The SLAM flap remains a robust option in the salvage setting, as its axial supply is typically spared from the microvascular rarefaction seen in previously irradiated fields, which can predispose random‐perforator flaps to failure and necrosis [13]. This axial blood supply is typically resilient to the microvascular changes induced by prior radiation therapy [14, 15], conferring resistance to radiation effects and ensuring viability even when subsequent postoperative radiation therapy is required [16, 17] This minimizes the risk of impaired wound healing and flap loss common in compromised fields.

## 3 Outcomes

Viability rates, extrapolated from the largest series of islanded FAMM variants, are consistently high. Reported partial/total necrosis rates are as low as 2.2% [18]. A literature review of 15 reconstructed SLA‐variant flaps reported no cases of necrosis (Table 2).

**TABLE 2:** Studies of SLAMM (superior labial artery musculomucosal flap) and rSLAM (reverse superior labial artery mucosal flap).

Tranchito [8]	Buccal (*n* = 1)	rSLAM	Nil
Güven [7]	Floor of mouth (*n* = 5)	SLAMM	Contracture (*n* = 1)
Momoh [6]	Nasal vestibule (*n* = 1)	rSLAM	Nil
Kashiwa [19]	Nasal ala (*n* = 4), nasal floor (*n* = 2), columella (*n* = 2)	SLAMM (*n* = 5) rSLAMM (*n* = 2), bipedicled SLAMM (*n* = 1)	Nil (*n* = 4), contracture (*n* = 1), temporary hypoesthesia (*n* = 1), flap drooping requiring repair (*n* = 2)

### 3.1 Reconstructive Fallback

Should flap compromise occur, several fallback strategies can be utilized. If the SLAM flap was intended as the internal liner for a composite reconstruction, immediate salvage can be achieved by folding a portion of the external fasciocutaneous skin paddle to provide internal coverage; however, this results in a bulkier, non‐mucosalized surface. Alternatively, a split‐thickness or full‐thickness skin graft can be used to line the internal surface, though these are prone to contracture and lack of pliability of the SLAM flap.

Finally, for smaller defects, a contralateral upper lip SLAM flap or a standard buccal‐based FAMM flap can be harvested, provided the respective donor site and FA remain viable. Because the SLAM flap is harvested from the upper lip to reconstruct the lower lip, the upper lip mucosal donor site is divided into distinct right and left vascular territories. If an initially raised ipsilateral SLAM flap experiences vascular compromise, the contralateral upper lip donor territory remains entirely uninjured. The remaining contralateral SLA provides a reliable, independent axial blood supply with sufficient length to allow the contralateral flap to be transposed across the midline to reach the lower lip defect.

### 3.2 Functional and Aesthetic Outcomes

Outcomes for reconstructions using FAMM flaps, which share the anatomic and functional characteristics of the SLAM flap, demonstrate high success rates in achieving functional rehabilitation. Functional outcomes confirm that using these flaps contributes positively to oral rehabilitation. Patients achieve high rates of normal speech articulation (90.9%) and maintain the ability to tolerate a regular diet (86.3%) [20]. Normal mouth opening, function (including the ability to pout) [21], and favorable aesthetics results (rated as excellent by 77.3% of patients in related FAMM series) [20] are maintained at 12‐month follow‐up. Additionally, partial recovery of lip sensation has been observed at 12 months postoperatively, a significant functional and aesthetic benefit [21]. In cases where primary closure of the donor site would lead to significant vertical shortening or distortion of the upper lip aesthetics, the donor defect may be left to heal by secondary intention to preserve the native craniocaudal height and natural contour of the upper lip.

## 4 Conclusion

The SLAM flap is a highly reliable, pedicled axial flap and primary option for lower lip reconstruction. Though limited by size, its greatest utility lies in providing superior mucosal tissue quality that is functionally unmatched by free flap transfers. The flap's robust supply from the SLA makes it a first‐line choice for the lower lip mucosal lining in oncologic defects and the preferred option as the internal liner in all major composite reconstructions requiring external bulk (e.g., RFFF). The SLAM flap provides an optimal mucosal interface, aids oral competence, and avoids the long‐term debulking often associated with folding fasciocutaneous flaps.

## Ethics Statement

As part of the routine surgical consent process, patients provided written informed consent for the collection of clinical data and photographs to be used for academic, research, and educational purposes. All patient identifiers have been removed to protect confidentiality in accordance with the Declaration of Helsinki.

## Conflicts of Interest

The authors declare no conflicts of interest.

## Data Availability

Data sharing is not applicable to this article as no datasets were generated or analysed during the current study.
